# Cinnabar-Induced Subchronic Renal Injury Is Associated with Increased Apoptosis in Rats

**DOI:** 10.1155/2015/278931

**Published:** 2015-01-06

**Authors:** Ying Wang, Dapeng Wang, Jie Wu, Bohan Wang, Xianhui Gao, Liangjun Wang, Honglin Ma

**Affiliations:** Department of Occupational and Environmental Health, School of Public Health, Liaoning Medical University, Jinzhou, Liaoning 121001, China

## Abstract

The aim of this study was to explore the role of apoptosis in cinnabar-induced renal injury in rats. To test this role, rats were dosed orally with cinnabar (1 g/kg/day) for 8 weeks or 12 weeks, and the control rats were treated with 5% carboxymethylcellulose solution. Levels of urinary mercury (UHg), renal mercury (RHg), serum creatinine (SCr), and urine kidney injury molecule 1 (KIM-1) were assessed, and renal pathology was analyzed. Apoptotic cells were identified and the apoptotic index was calculated. A rat antibody array was used to analyze expression of cytokines associated with apoptosis. Results from these analyses showed that UHg, RHg, and urine KIM-1, but not SCr, levels were significantly increased in cinnabar-treated rats. Renal pathological changes in cinnabar-treated rats included vacuolization of tubular cells, formation of protein casts, infiltration of inflammatory cells, and increase in the number of apoptotic tubular cells. In comparison to the control group, expression of FasL, Fas, TNF-*α*, TRAIL, activin A, and adiponectin was upregulated in the cinnabar-treated group. Collectively, our results suggest that prolonged use of cinnabar results in kidney damage due to accumulation of mercury and that the underlying mechanism involves apoptosis of tubular cells via a death receptor-mediated pathway.

## 1. Introduction

Cinnabar is a naturally occurring mercuric sulfide (HgS ≥ 96%) [1]. Cinnabar powder has been used as an important ingredient in traditional Chinese medicines and in Indian Ayurvedic medicines [2] for more than 2000 years. According to Chinese Pharmacopoeia (2010), approximately 5% of Chinese patented medicines contain cinnabar. Cinnabar-containing preparations have been used extensively for treatment of chronic ailments, including syphilis, high fever, pneumonia, insomnia, nervous disorders, deafness, and paralysis of the tongue [3–6]. Although cinnabar is generally thought to be slight toxic because HgS is almost insoluble in water, adverse reactions to cinnabar or cases of intoxication have occasionally been reported in the clinical literature [7–10]. These toxicities primarily result from inappropriate usage, such as excessive and long-term dosage [10]. The adverse effects of cinnabar are due to the presence of mercury [11, 12]. The kidneys are the primary target organ where inorganic mercury is taken up, is accumulated, and expresses toxicity [13]. The distribution of mercury within the body resulting from cinnabar absorption is similar to that of inorganic mercury, and the highest concentration of mercury is found in the kidney [14–16]. Excessive or long-term use of cinnabar or cinnabar-containing preparations can result in renal dysfunction due to accumulation of mercury [10]. Although several studies have reported the renal toxicity of cinnabar, the underlying mechanism associated with this toxicity remains unclear.

It is well-known that mercury compounds are nephrotoxicants. The toxic effect of mercury results from tight binding to sulfhydryl, carboxyl, and phosphoryl groups, which are abundant in proteins and polypeptides of organisms. Within the kidney, the proximal tubule is the most vulnerable segment of the nephron to the toxic effects of mercury [13]. Oxidative stress and apoptosis have been shown to be important mechanisms in mercury-induced renal toxicity through studies using mercuric chloride as nephrotoxicant [17–20]. It is well known that the specific chemical forms of mercurial compounds are important for determining their toxicity. A previous study has shown that cinnabar-induced renal injury may be associated with oxidative stress [21]. However, it remains unclear whether apoptosis plays a role in cinnabar-induced renal injury.

To address this question, we induced subchronic renal toxicity resulting from cinnabar administration in rats. Hg content was analyzed to confirm mercury accumulation in the kidney, and markers of renal injury and pathology were examined to evaluate renal injury. Levels of apoptosis in the kidney were assessed by hematoxylin and eosin (HE) staining, transmission electron microscopy, and the TUNEL assay. Furthermore, we measured the expression of cytokines in the kidney using an antibody array in order to explore activation of specific apoptotic pathways. Our results suggest that cinnabar-induced renal injury is associated with increased apoptosis of tubular cells and that death receptors play a primary role in activation of this apoptotic pathway.

## 2. Materials and Methods

### 2.1. Animals

Sprague-Dawley rats (160 ± 20 g) were obtained from Vital River Laboratory Animal Technology Co. Ltd. (Beijing, China). All animal experiments were performed with the approval of the Ethical Committee of Liaoning Medical University (number Gb05130156). Rats were housed in a temperature-controlled (22 ± 1°C) room, maintained on a 12-hour light/dark cycle, and held in quarantine for 1 week prior to the initiation of dosing. With the exception of overnight fasts before scheduled necropsies, rats were allowed free access to food and water throughout the study.

### 2.2. Rat Cinnabar Subchronic Poisoning Model

After release from quarantine, male and female rats were randomly assigned to the control group and the cinnabar group (8 male and 8 female in each group). Rats in the cinnabar group were treated with cinnabar (96% HgS, De-Chang-Xiang Drug Company, Guiyang, GuiZhou, China) at dose of 1 g/kg/day in 0.5% sodium carboxymethyl cellulose solution by oral administration (gavage), which corresponds to 20 times of clinical allowable limit of cinnabar according to Chinese Pharmacopoeia 2010 edition. Control rats received an equal volume (10 mL/kg/day) of solvent. Half of the rats of each sex in each group underwent necropsy after consecutive dosing for 8 weeks or 12 weeks. The dose of cinnabar and the period of treatment are based on the literatures [22, 23]. During the exposure period, clinical symptoms, including but not limited to changes in behavior, activity, skin, fur, excretions, mortality, and other clinical signs of toxicity, were observed once a day, and body weight was weighed weekly. After the rats were sacrificed, blood and urine samples were collected for estimation of Hg content and parameters related to nephrotoxicity, and kidney tissues were collected for pathological examination or stored at −80°C for other experiments.

### 2.3. Determination of Mercury Levels in the Kidney

The mercury standard solution (1000 *μ*g/mL, National Analysis Center for Iron and Steel, Beijing, China) was diluted with 0.5 g/L potassium dichromate to 10 *μ*g/mL. The resulting solution was then diluted with 5% nitric acid to 100 *μ*g/L and was finally diluted with ultrapure water to generate a series of standard solutions (0 *μ*g/L, 0.5 *μ*g/L, 1 *μ*g/L, 5 *μ*g/L, 10 *μ*g/L, and 15 *μ*g/L).

Renal mercury (RHg) was measured by hydride generation atomic fluorescence spectrometry. Renal samples (0.3 g) were placed into a tube special for digestion and digested with a mixture of 6 mL nitric acid and 2 mL hydrogen peroxide. The samples were predigested overnight at room temperature, followed by digestion in a microwave oven (CEM, Matthews, North Carolina, USA) as follows: heated to 120°C within 5 min and lasted for 1 min; heated to 160°C within 5 min and lasted for 5 min; heated to 170°C within 3 min and lasted for 10 min; and maximum power was 1600 W. Digested samples were heated at 90°C for 90 min to remove the acids and were then diluted with ultrapure water to a final volume of 50 mL. Hg content of each sample was measured using a hydride generator (MHS15, PerkinElmer, Massachusetts, USA) and an atomic fluorescence photometer (AFS-230E, Kechuang Haiguang, Beijing, China) under the following conditions: lamp current, 10 mA; photomultiplier tube electric voltage limit, –250 V; atomizer height, 10 mm; carrier gas flow rate, 500 mL/min; shielding device flow rate, 1.0 L/min. A standard curve was prepared using the mercury standard series described above. Values were obtained by reading the peak area with a 10 sec read time and a 1 sec delay time. The blank discriminant value was 2. Each sample was read 3 times. Standard addition and recovery experiments were conducted to control for the quality of this analysis method.

### 2.4. Determination of Mercury Levels in Urine

The preparation of mercury standard solution was the same as the Section 2.3. Urinary mercury (UHg) was measured by hydride generation atomic absorption spectrometry. Urine samples were treated as follows. First, a 2 mL sample was placed into a colorimetric tube with a plug, and ultrapure water was added to a volume of 10 mL and blended. Next, 2 mL potassium permanganate (50 g/L) and 1 mL concentrated sulfuric acid were added to the solution, which was then blended and incubated for 5 min at room temperature. The mixture was then heated in a water bath at 50°C for 2 h. Hydroxylamine hydrochloride solution (200 g/L) was added dropwise with agitation, and the resulting solution was incubated for 30 min in the tube without the plug. Next, 5 mL hydrochloric acid (diluted with an equal volume ultrapure water) was added to the mixture. Finally, ultrapure water was added to a final volume of 50 mL, and the mixture was blended. Hg content was measured using a hydride generator (MHS15, PerkinElmer, Massachusetts, USA) and an atomic absorption spectrometer (AA800, PerkinElmer, Massachusetts, USA) under the following conditions: sample, 10 mL; reducing agent, 2 mL 3% NaBH_4_; carrier gas, argon; wavelength, 253.7 nm; lamp current, 6 mA; slit width, 0.7 nm. A standard curve was prepared using the mercury standard series described above. Values were obtained through reading peak height with a 10 sec read time. Each sample was read 3 times. Standard addition and recovery experiments were conducted to control for the quality of this analysis method.

### 2.5. Clinical Chemistry

Blood and urine samples were collected from sacrificed rats. The levels of kidney injury molecule 1 (KIM-1) in urine were detected using ELISA kits (Abcam, Cambridge, UK) according to the manufacturer's instructions. Concentrations of blood serum creatinine (SCr) were determined using an automatic analyzer (7180, Hitachi, Tokyo, Japan).

### 2.6. Kidney Histopathology

Kidney tissues were fixed in 10% buffered formalin and embedded in paraffin. Sections (5 *μ*m) were stained with hematoxylin and eosin (HE) to evaluate histopathological injury by light microscopy. For ultrastructure evaluation, formalin-fixed kidney pieces were transferred to phosphate-buffered 2.5% glutaraldehyde and embedded in epoxy resin. Thin sections (50 nm) were collected on copper grids, stained with uranyl acetate and lead citrate, and examined with a transmission electron microscope (JEM-1200EX, JEOL, Tokyo, Japan).

### 2.7. TUNEL Assay

Apoptosis in kidney tissue sections (5 *μ*m) was analyzed using the DeadEnd colorimetric TUNEL system (Promega, Madison, WI, USA) according to the manufacturer's instructions. Cells stained brown were considered to be positive for apoptosis. Five fields (400x) were randomly selected for each section, and the number of positive cells and total cells were counted in each field. These values were used to calculate the apoptotic index (AI), defined as the percentage of positive cells.

### 2.8. Measurement of Cytokines

Cytokine levels in kidneys from rats sacrificed at 12 weeks were measured using a biotin label-based rat antibody array (RayBio, AAR-BLM-1, RayBiotech, Norcross, GA, USA). First, approximately 10 mg of renal tissue was homogenized in 0.4 mL lysis buffer and centrifuged at 13000 rpm for 10 min. The supernatant was transferred to a dialyzer and dialyzed with 4000 mL PBS buffer (pH 8) at 4°C. Change the PBS buffer and dialyze again. Allow 3 h for each dialysis step; stir gently. Protein concentrations of the resulting tissue lysates were measured using a BCA kit (Pierce, Rockford, IL, USA). Next, samples were labeled with biotin using a labeling reagent from the kit. After incubation at room temperature for 30 min, stop solution from the kit was added, and free biotin was removed using a spin column. The column was then centrifuged at 1000 ×g for 3 min to collect the sample. Each membrane was placed into the provided tray, blocking buffer was added, and the membrane was incubated at room temperature for 1 h. The blocking buffer was decanted from each container. The membranes were incubated with samples diluted in blocking buffer at 4°C with gentle shaking overnight. The membranes were then washed with wash buffer from the kit. Following washing, the membranes were incubated with 1 : 8000 diluted IRDye : emoji : 800CW-streptavidin (LI-COR, Lincoln, NE, USA) in the absence of light at room temperature with gentle agitation for 2 h. Finally, bound streptavidin was detected using an odyssey scanner (LI-COR, Lincoln, NE, USA), and the images were analyzed with the RayBio analysis tool.

### 2.9. Statistical Analysis

Statistical analysis was performed using Student's *t*-test. The data were expressed as mean ± standard deviation (SD), and *P* values less than 0.05 were considered statistically significant.

## 3. Results

### 3.1. General Toxic Effects of Cinnabar

To study the subchronic renal toxicity of cinnabar, rats were administered 1 g/kg/day cinnabar orally for 8 or 12 consecutive weeks. All rats appeared to be in good condition over the course of the experiment. No abnormalities in diet, weight gain, or activity of the cinnabar-treated rats were observed in comparison with the controls. The only observed difference between the two groups was the presence of red feces in the experimental group, which contained unabsorbed cinnabar. There were no significant differences in kidney to body weight ratio between the two groups at 8 weeks or 12 weeks.

### 3.2. Mercury Concentrations in Urine and Kidney

**Figure 1:**
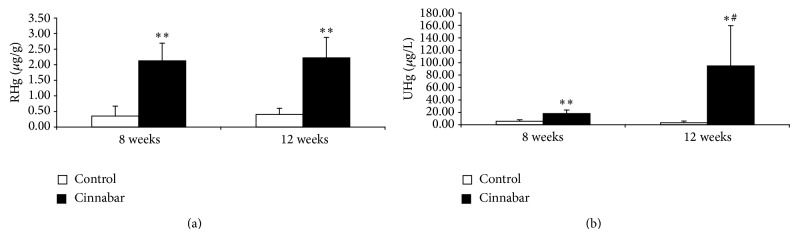
Renal and urinary mercury levels are elevated in cinnabar-treated rats. Rats were dosed with cinnabar (1 g/kg/day) for 8 weeks or 12 weeks. Levels of (a) renal mercury (RHg) and (b) urinary mercury (UHg) were analyzed. All values are expressed as mean ± SD (*n* = 6). ^*^ *P* < 0.05, ^**^ *P* < 0.01, compared with the control group. ^#^ *P* < 0.05, compared with cinnabar group sacrificed at 8 weeks.

Urinary mercury is an ideal biomarker for long-term exposure to mercury. The content of renal mercury directly reflects the accumulation of mercury in the kidney. To confirm the accumulation of mercury in the kidney after exposure of rats to cinnabar, we analyzed RHg and UHg levels (Figure 1). Compared with the control group, both RHg and UHg levels increased at 8 weeks in the cinnabar group (*P* < 0.01). At 12 weeks, UHg levels in the cinnabar group increased to an even greater degree (*P* < 0.05 versus the control, *P* < 0.05 versus 8 weeks), while RHg levels were similar to those at 8 weeks (*P* < 0.01 versus the control, *P* > 0.05 versus 8 weeks).

### 3.3. Effect of Cinnabar on Renal Function

**Figure 2:**
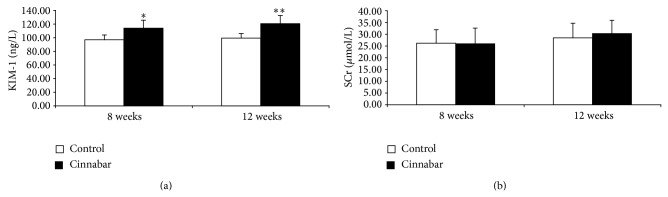
Changes in markers of renal function in cinnabar-treated rats. Rats were dosed with cinnabar (1 g/kg/day) for 8 weeks or 12 weeks. Levels of (a) urinary kidney injury molecule 1 (KIM-1) and (b) blood serum creatinine (SCr) were measured. All values are expressed as mean ± SD (*n* = 6). ^*^ *P* < 0.05, ^**^ *P* < 0.01, compared with the control group.

SCr is a classic marker of glomerular dysfunction, and urinary KIM-1 is a sensitive marker of proximal tubule injury resulting from a variety of chemical agents. To identify signs of injury to renal function caused by cinnabar, we measured urinary KIM-1 and SCr (Figure 2). Compared with the control group, KIM-1 was increased at both 8 weeks and 12 weeks in the cinnabar group (*P* < 0.05, *P* < 0.01, resp.). In contrast, levels of SCr did not increase significantly.

### 3.4. Effect of Cinnabar on Renal Histopathology

**Figure 3:**
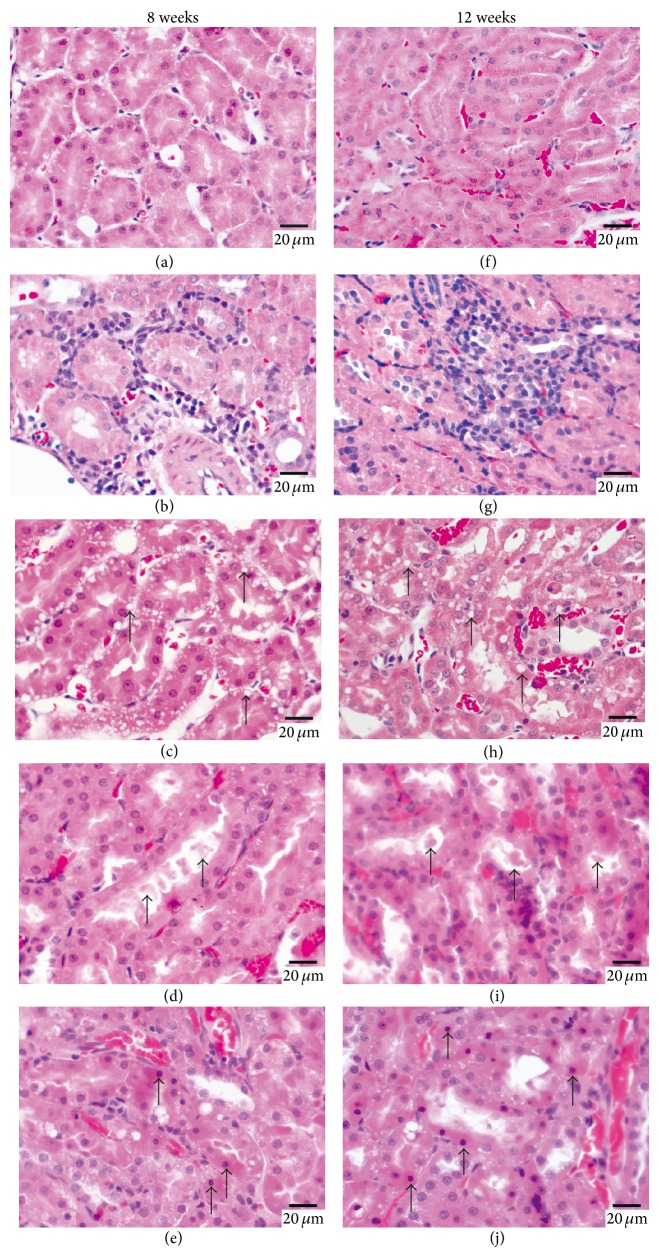
Representative images of rat kidney histopathology in cinnabar-treated rats. Rats were dosed with cinnabar (1 g/kg/day) for 8 weeks or 12 weeks. Renal sections were stained with hematoxylin and eosin (HE). Representative images are shown from (a), the control group, 8 weeks (b–e), the cinnabar group, 8 weeks (f), the control group, 12 weeks (g–j) the cinnabar group, 12 weeks. (a) and (f) show the normal histology of the kidney. (b) and (g) show infiltration of inflammatory cells. (c) and (h) show vacuolization of tubular cells (indicated by arrows), (d) and (i) show protein casts in tubules (indicated by arrows), and (e) and (j) show apoptotic cells (indicated by arrows) in cinnabar-treated animals. Scale bar = 20 μm.

**Figure 4:**
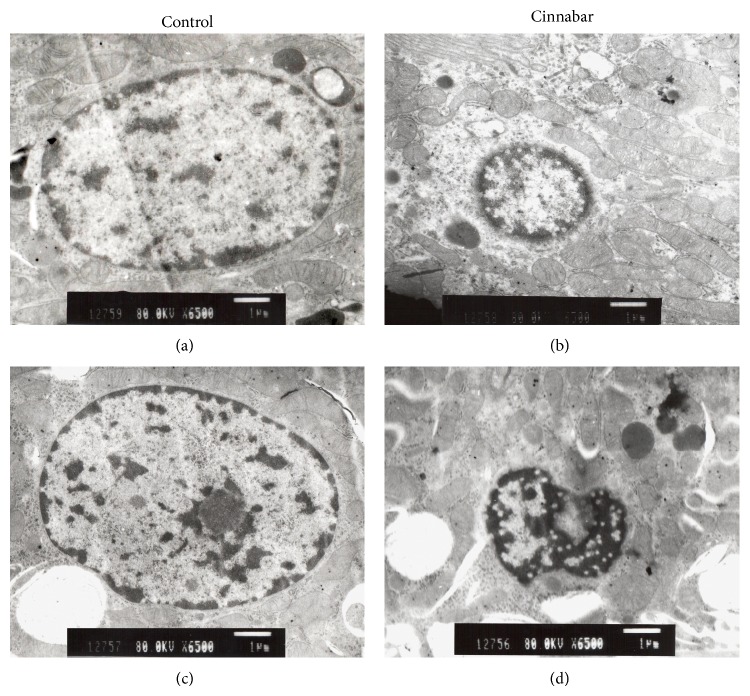
Electron microscope images of rat kidney sections. Rats were dosed with cinnabar (1 g/kg/day) for 8 weeks or 12 weeks. (a), the control group, 8 weeks; (b), the cinnabar group, 8 weeks; (c), the control group, 12 weeks; (d) the cinnabar group, 12 weeks. (a) and (c) show a normal cell nucleus. (b) and (d) show an apoptotic cell nucleus. Scale bar = 1 μm.

To further assess renal tissue injury caused by cinnabar, we performed renal pathological examination. Kidney sections stained with HE were observed under light microscopy (Figure 3) after 8 weeks of treatment (Figures 3(a)–3(e)) and 12 weeks of treatment (Figures 3(f)–3(j)). No lesions were found in samples in the control group (Figures 3(a) and 3(f)). In the cinnabar group, observed pathological changes included infiltration of inflammatory cells (lymphocytes, monocytes, and plasmocytes) (Figures 3(b) and 3(g)), vacuolization of tubular cells (Figures 3(c) and 3(h)), and the presence of protein casts in the tubules (Figures 3(d) and 3(i)). Moreover, cells that met the morphological criteria for apoptosis (i.e., nuclear pyknosis and hyperchromatic cytoplasm) were also observed in the renal tubules of cinnabar-treated animals (Figures 3(e) and 3(j)). To confirm the presence of these apoptotic cells, samples were further examined by transmission electron microscopy (Figure 4). In kidney sections from cinnabar-treated rats, shrunken karyons with chromatin condensation were observed (Figures 4(b) and 3(d)), consistent with the presence of apoptotic cells.

**Figure 5:**
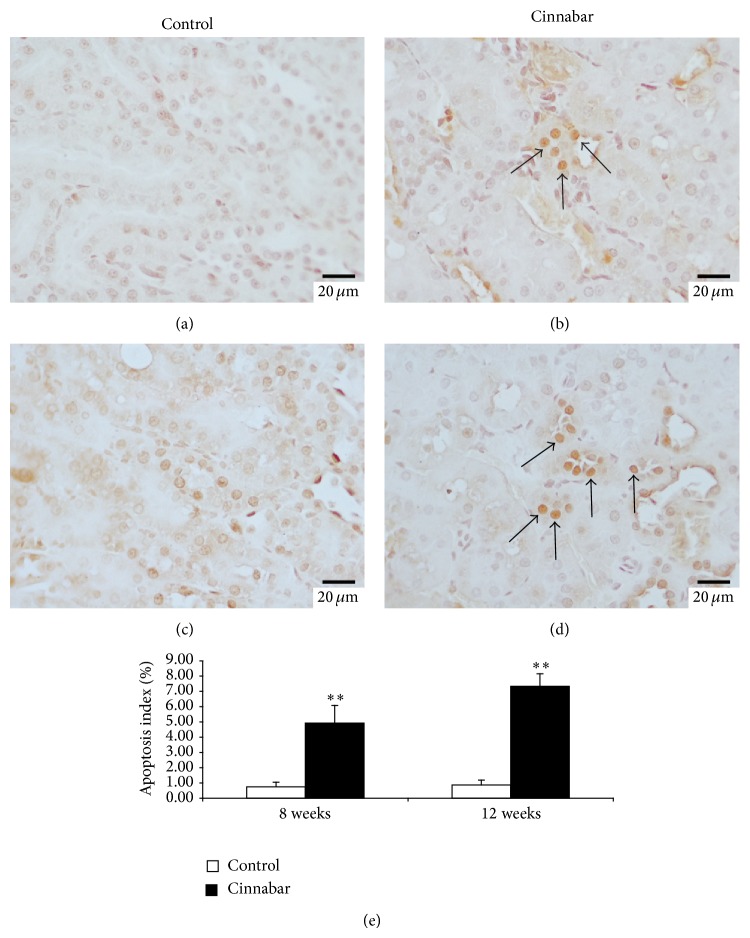
Analysis of apoptosis using the TUNEL assay in kidney samples from cinnabar-treated rats. Rats were dosed with cinnabar (1 g/kg/day) for 8 weeks or 12 weeks. Apoptosis in kidney sections was detected using an* in situ* TUNEL assay. TUNEL-positive apoptotic cells are indicated by brown staining (see arrows). (a) Control group at 8 weeks. (b) Cinnabar group at 8 weeks. (c) Control group at 12 weeks. (d) Cinnabar group at 12 weeks. (e) Bar graph of apoptosis index. Scale bar = 20 μm in (a–d). ^**^ *P* < 0.01, compared with the control group. (*n* = 6).

To further explore whether cinnabar-induced renal injury was associated with apoptotic cell death, we carried out an* in situ* TUNEL assay on renal sections (Figure 5). Cells stained brown are indicative of TUNEL-positive, apoptotic cells. In the control group, only a few positive cells were found (Figures 5(a) and 5(c)). In contrast, a greater number of apoptotic cells were observed in renal tubules of rats from the cinnabar group (Figures 5(b) and 5(d)). Furthermore, the apoptotic index was increased in the cinnabar group at both 8 and 12 weeks (*P* < 0.01) (Figure 5(e)). Collectively, these results indicate that increased apoptosis is associated with renal injury caused by cinnabar.

### 3.5. Effect of Cinnabar on Expression of Apoptosis-Related Cytokines in Rat Kidney

**Figure 6:**
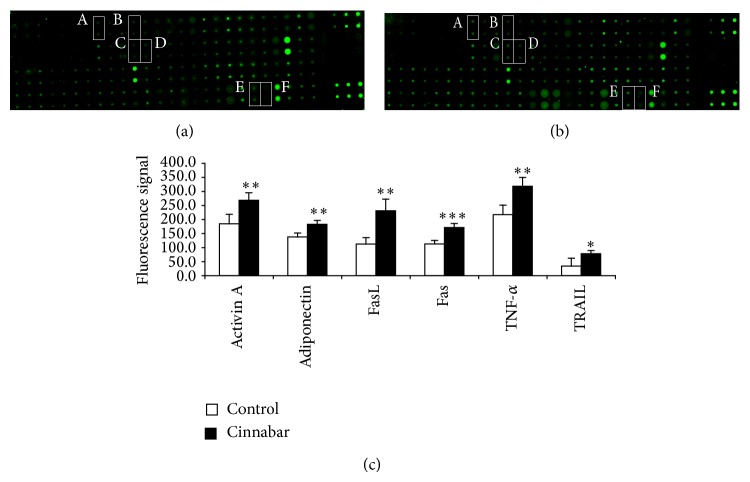
Change in expression of cytokines associated with apoptosis in rat kidney. Rats were dosed with cinnabar (1 g/kg/day) for 12 weeks. Expression of cytokines associated with apoptosis in the kidney was analyzed by antibody array (RayBio, AAR-BLM-1). (a) Representative image from the control group. (b) Representative image from the cinnabar group. (c) Bar graph showing cytokines expression in both groups. (A) Activin A, (B) adiponectin, (C) Fas ligand (FasL), (D) Fas, (E) TNF-*α*, and (F) TNF-related apoptosis-inducing ligand (TRAIL). ^*^ *P* < 0.05, ^**^ *P* < 0.01, ^***^ *P* < 0.001, compared with the control group. (*n* = 4).

To further explore the mechanism of cinnabar-induced apoptosis, we next measured the expression of cytokines associated with apoptosis using an antibody array (Figure 6). In comparison to the control group, levels of Fas ligand (FasL, TNFSF6) and Fas (TNFRSF6) significantly increased in the cinnabar group (*P* < 0.01, *P* < 0.001, resp.), in addition to expression of tumor necrosis factor-alpha (TNF-*α*) and TNF-related apoptosis-inducing ligand (TRAIL and TNFSF10) (*P* < 0.01, *P* < 0.05, resp.). Levels of activin A and adiponectin were also elevated in the cinnabar group (*P* < 0.01 and *P* < 0.01, resp.).

## 4. Discussion

Cinnabar, an important traditional Chinese medicine, has been widely used in the clinic. According to the Chinese Pharmacopoeia (2010 edition), a daily allowable amount of cinnabar is 0.1–0.5 g, and cinnabar should not be taken excessively or for long period, but no specific period is formulated. Although cinnabar is less toxic than HgCl_2_ and MeHg [7–9, 24], its potential toxic effects on the kidney cannot be ignored if taken over a prolonged period, especially at higher doses, due to excessive accumulation of mercury in the kidney [25]. In this study, rats were dosed with 1 g/kg/day cinnabar (corresponding to 20 times of clinical allowable limit) for 8 weeks or 12 weeks, and cinnabar-induced subchronic renal injury in rats was assessed, and the underlying mechanism was investigated.

Our results demonstrate that both UHg and RHg levels were significantly increased in cinnabar-treated rats, confirming absorption of cinnabar. Hg was partially excreted in urine and partially accumulated in the kidney. UHg is an ideal biomarker for long-term exposure to mercury and is also a good indicator of body burden [26]. Although the cumulative intake of cinnabar continued to increase over time, the RHg levels at 12 weeks were similar to those at 8 weeks because levels of UHg were still on the increase. Urinary KIM-1, an indicator of renal proximal tube injury, has been shown to be elevated at an early stage of injury, and increased levels are sustained throughout the progression of cell injury to proliferation and regeneration [27]. SCr is a classical, though not sensitive, marker of glomerular function. In this study, urinary KIM-1 levels in the cinnabar-treated group were significantly increased, but SCr levels did not significantly change. Mercury primarily damages the renal tubules and has also been shown to damage the glomerulus in severe cases [28]. Examination of renal pathology revealed the presence of vacuoles in tubular cells and protein casts in tubules from cinnabar-treated rats, consistent with a previous study [23]. Moreover, we observed mild proliferation of mesangial matrix in cinnabar groups (the result was not shown in this paper). In contrast, other studies [7–9, 21, 29] have reported varying extents of cinnabar-induced kidney lesions resulting from different dosages and durations of treatment. In general, cinnabar primarily injured the renal tubules, which probably related to the distribution and localization of mercury in the kidney. The segments of the proximal tubule are the primary sites where mercuric ions are taken up and accumulated [13].

Apoptosis (also known as programmed cell death) is considered to be an ongoing normal event in the control of cell populations and is a highly regulated and crucial process found in all multicellular organisms [30, 31]. However, apoptosis can also be induced by a variety of xenobiotics, including toxic metals [32–34] and drugs [35, 36]. Abnormal apoptosis can result in a disruption of tissue homeostasis and function and is associated with many diseases [37], including numerous renal diseases in which the severity of renal injury is below the threshold for the development of necrosis [38]. Previous studies have shown that herbs may cause nephrotoxicity by induction of apoptosis [35] and that nephrotoxicity from heavy metals (such as cadmium, mercury, and lead) also involves apoptosis [32, 39, 40]. Cinnabar is a traditional Chinese medicine that contains mercury, and in this study we observed an increase in apoptosis in kidney tissues from cinnabar-treated rats, which is consistent with previous reports demonstrating that inorganic mercury accumulates in the proximal tubules of the kidney and causes renal apoptosis [13]. Moreover, use of the TUNEL assay further confirmed an increase in the apoptosis index in kidney tissue from cinnabar-treated rats compared with control rats, providing further support for the hypothesis that activation of apoptosis in renal tubular cells was associated with cinnabar-induced renal injury.

Apoptosis can be initiated by many signaling pathways, including stimulation of death receptors (DRs), such as FasR, TNFRs, and TRAILRs, by their respective ligands. The central execution machinery primarily consists of caspases, a family of cysteine proteases that function as a signaling cascade in the death process [41]. In this study, we found that expression of FasL, Fas, TNF-*α*, and TRAIL in the kidney was significantly increased in cinnabar-treated rats in comparison to control rats. In the kidney, FasL and TNF can be synthesized by both intrinsic renal cells and infiltrating leukocytes [42]. The tubular epithelial cell is the main source of intrinsic FasL [43]. Tubular cells are quite resistant to FasL-induced apoptosis under basal conditions [44], but become sensitized upon exposure to inflammatory cytokines, including TNF-*α* [43]. TRAIL, also known as APO2-L, induces apoptosis by binding to TRAILRs. In normal kidney tissue, TRAIL is expressed only in tubules and is absent from glomeruli [45]. Inflammatory cytokines, such as TNF, induce TRAIL expression in tubular cells [43, 46]. When normal cells are immersed in an inflammatory environment, TRAIL may induce parenchymal cell apoptosis [47]. In this study, we observed infiltration of inflammatory cells, such as lymphocytes, monocytes, and plasmocytes, into the renal tubulointerstitial space in cinnabar-treated rats. Thus, we hypothesize that the DR-mediated pathway plays a major role in apoptosis of renal tubular cells induced by cinnabar.

We also found that expression of activin A and adiponectin was markedly upregulated in cinnabar-treated rats. Activin A can inhibit cell proliferation and induce apoptosis in cultured proximal tubular cells [48]. Adiponectin, also called Acrp30 or GBP28, is a multifunctional cytokine that plays an important role in the regulation of inflammation and energy metabolism. The effect of adiponectin on apoptosis is controversial [49]. However, targeted disruption of adiponectin has been shown to protect against tubular cell apoptosis and to reduce inflammatory cell infiltration and proinflammatory molecule production in the kidney [50].

In summary, results from this study suggest that prolonged use of cinnabar can cause kidney damage resulting from accumulation of mercury. Kidney injury was reflected in increased urinary KIM-1 levels, vacuolization and apoptosis of tubular cells, protein cast formation, and infiltration of inflammatory cells. Furthermore, increased apoptosis of tubular cells was found to be associated with cinnabar-induced renal injury and appears to be mediated by the DR-dependent pathway.

## 5. Conclusions

Prolonged use of cinnabar can cause kidney damage resulting from accumulation of mercury. Furthermore, increased apoptosis of tubular cells was associated with cinnabar-induced renal injury and appears to be mediated by the DR-dependent pathway.
